# Kir6.1/K-ATP channel in astrocytes is an essential negative modulator of astrocytic pyroptosis in mouse model of depression

**DOI:** 10.7150/thno.77455

**Published:** 2022-09-11

**Authors:** Feng Li, Si-Yuan Jiang, Tian Tian, Wen-Jie Li, You Xue, Ren-Hong Du, Gang Hu, Ming Lu

**Affiliations:** 1 Jiangsu Key Laboratory of Neurodegeneration, Department of Pharmacology, Nanjing Medical University, 101 Longmian Avenue, Nanjing, Jiangsu 211166, P.R. China.; 2 Department of Pharmacology, Nanjing University of Chinese Medicine, 138 Xianlin Avenue, Nanjing, Jiangsu 210023, P.R. China.

**Keywords:** depression, astrocyte, Kir6.1, pyroptosis, NLRP3 inflammasome

## Abstract

**Rationale:** Astrocyte dysfunction is one of the important pathological mechanisms of depression. Stress contributes to the onset of depression. As metabolic stress sensor, Kir6.1-contaning K-ATP channel (Kir6.1/K-ATP) is prominently expressed in astrocytes. However, the involvement of Kir6.1/K-ATP channel in depression remains obscure.

**Methods:** Astrocyte-specific Kir6.1 knockout mice were used to prepare two mouse models of depression to explore the role of astrocytic Kir6.1/K-ATP channel in depression. Primary astrocytes were cultured to reveal the underlying mechanism for Kir6.1-regulated astrocytic pyroptosis.

**Results:** We identified that chronic stress reduced the astrocytic Kir6.1 expression in hippocampus of mice. We further observed astrocyte-specific knockout of Kir6.1 induced depressive-like behaviors in mice. Moreover, we found that astrocytic Kir6.1 deletion increased NLRP3-mediated astrocytic pyroptosis in response to stress. Mechanistically, Kir6.1 associated with NLRP3, and this interaction prevented the assembly and activation of NLRP3 inflammasome, thereby inhibition of astrocytic pyroptosis. More importantly, VX-765, an effective and selective inhibitor for NLRP3 inflammasome, could reverse the astrocytic pyroptosis and rescue the deterioration of behaviors in astrocytic Kir6.1 knockout mice.

**Conclusions:** Our findings illustrate that Kir6.1/K-ATP channel in astrocytes is an essential negative modulator of astrocytic pyroptosis and plays a crucial role in depression and suggest that astrocytic Kir6.1/K-ATP channel may be a promising therapeutic target for depression.

## Introduction

Depression is a major cause of disability that affects approximately over 300 million people worldwide 1, 2. Although various hypotheses have been involved in this disease, including monoamine neurotransmitter disturbance, hypothalamic/pituitary/adrenal axis disorder, neurotrophic factor deficiency, neuro-inflammation, and immune dysfunction, the exact pathophysiological mechanism of depression is still elusive 3-5. Accumulating evidence has demonstrated that astrocytes are highly correlated to the pathophysiological process of depression 6. First, the number of astrocytes have been found to be reduced in the depressed patients and in animal models of depression 7, 8. Second, antidepressant treatment can increase the gene expression and proliferation of astrocytes 9, 10. Third, pharmacologic glial deletion induces depressive-like behaviors of mice 11. However, the cause of astrocytic loss in the pathogenesis of depression remains unknown.

Pyroptosis is a novel form of programmed cell death executed by gasdermin (GSDM) family proteins 12. It is characterized by NLRP3 activation, GSDMD protein cleavage, and the cell membrane pore formation 13. The GSDMD-N-terminal domain fragments is generated from GSDMD protein cleaved by caspases through NLRP3 inflammasome, and then rapidly translocate to the plasma membrane to form an active membrane pores, leading to pyroptosis 14. The activation of pyroptosis has been reported in various central system diseases, such as multiple sclerosis 15, Parkinson's disease 16, Alzheimer's disease 17, and depression 18. Thus, inhibition of cell pyroptosis has been thought to be a promising therapeutic strategy for these diseases.

ATP-sensitive potassium (K-ATP) channels are the unique potassium channels coupling cell metabolism to cell membrane potential 19. They are composed of pore-forming Kir6.x (6.1 or 6.2) subunits and sulfonylurea receptor subunits, regulated by intracellular ATP and ADP concentrations. Our previous study found that activation of K-ATP channel stabilized HPA axis disturbance via inhibition of neuro-inflammation 20. In the brain, Kir6.2 is mainly expressed in neurons, and Kir6.2 knockout aggravated depression-like behaviors via promoting the CA3 neurons death in mouse model of depression 21. As a stress sensor, Kir6.1 is predominantly expressed in astrocytes 22 and regulates astrocyte-mediated neuron-inflammation 23. But, the role of astrocytic Kir6.1/K-ATP channel in depression is still unclear. In the present study, we aimed to explore the exact effect of astrocytic Kir6.1/K-ATP channel in depression and to reveal the underlying mechanism for Kir6.1-regulated astrocytic pyroptosis.

## Materials and methods

### Experimental animals

Astrocyte-specific Kir6.1 knockout mice (Kir6.1^loxp/loxp^ GFAP^Cre^, CKO) on a C57/B6 background were produced by breeding the Kir6.1^floxed^ mice with GFAP-driven Cre recombinase mice 24. Kir6.1^loxp/loxp^ littermates (WT) were used as controls. Mice were bred and maintained in the Animal Resource Centre of the Faculty of Medicine, Nanjing Medical University. Mice are free access to food and water in a room with a 12 : 12 h light/dark cycle and an ambient temperature of 20 °C ± 2 °C. All animal procedures were performed in strict accordance with the guideline of the Institutional Animal Care and Use Committee (IACUC) of Nanjing Medical University.

### Chronic unpredictable mild stress (CUMS) procedure

Both genotypic mice (male, 8-12 weeks) were individually housed and subjected to 5 weeks of stressors, which were mild and unpredictable in nature, duration, and frequency. Stressors included inversion of day/night light cycle for 24 h, soiled cage bedding for 12 h, 45° tilted cage for 18 h, restraint for 4 h, food deprivation for 24 h, water deprivation for 12 h, cage shaking for 10 min, tail nip for 1 min (1 cm from the end of the tail). Two different stressors were used in one day, and the same stressor was not scheduled in two adjacent days. Sucrose preference and body weight of each animal were evaluated weekly until the end of the CUMS.

### Chronic social defeat stress (CSDS)

The CSDS paradigm was conducted as previously described 25. Adult male CD1 mice with the age of 20-30 weeks old and the body weight over 40 g were housed in the defeat cages and used as aggressors in subsequent social defeat experiments. Briefly, CKO and WT mice were defeated by a novel CD-1 aggressor for 10 min daily over 10 consecutive days. The defeated mouse was subjected to continuous psychological stress from a CD-1 mouse through a clear perforated divider allowing for visual, olfactory, and auditory contact in a shared home cage for the next 24 h after defeat. Control mice were housed in their home cages and allowed to explore the empty defeat cages for 10 min each day. CKO mice were injected intraperitoneally with VX765 (100 mg/kg, S2228, Selleck, China) dissolved in DMSO once daily for 10 consecutive days, beginning immediately after the first challenge and continuing until the last day of challenge.

### Social interaction test

The social interaction test was used to determine social avoidance behavior. Briefly, in the first “No target” trial, an empty cage was placed in the interaction zone. In the second “Target” trial, a novel CD-1 mouse was placed inside the cage in the interaction zone. Each mouse was allowed to freely explore the environment for 5 min with its movement tracked. The mouse was habituated to the chamber in the absence of a CD-1 mouse for 5 min prior to the test. After each trial, the equipment was cleaned with 75% ethanol to remove olfactory cues. Each trial lasted for 5 min, and the duration time in the interaction zone spent by the test mouse was individually measured using Ethovision video tracking software (Noldus Technology). Social interaction ratio was calculated according the following formula: Social interaction ratio = time spent in the interaction zone in the presence of target/time spent in the interaction zone in the absence of target × 100%.

### Sucrose preference test

Mice were water deprived for 12 h, and then were allowed to drink from two bottles for 10 h: one contained 1% sucrose solution and the other only tap water. To prevent the possible effect of side-preference in drinking behavior, the positions of the bottles in the cage were switched after the first 5 h. The consumption of tap water, sucrose solution, and total intake of liquids was estimated simultaneously in the control and experimental groups by weighing the bottles. The preference for sucrose was measured as a percentage of the consumed sucrose solution relative to the total amount of liquid intake.

### Tail suspension test (TST)

Mice tails were wrapped with tape from the base to the tip, covering about 4/5 of its length, and fixed upside down on the hook. The immobility time of each mouse was recorded over a 6 min period. Mice were considered immobile only when they hung passively and completely motionless. The duration of immobility of the tail suspended mice during the last 4 min was measured with TailSuspScan™ (Clever Sys Inc., USA).

### Forced swim test (FST)

Mice were individually forced to swim in an open cylindrical container (diameter, 15 cm; height, 25 cm), containing 14 cm of water at room temperature (about 20 ± 2 °C) for 6 min. A mouse was judged to be immobile when it floated in an upright position, and made only small movements to keep its head above water. The duration of immobility was recorded during the last 4 min of the 6 min testing period by TailSuspScan™ (Clever Sys Inc., USA).

### Immunohistochemistry and quantification of staining

After perfusion, brain samples were collected and post-fixed in 4% paraformaldehyde solution at 4 °C overnight. The brains were transferred to 20% sucrose in phosphate-buffered saline (PBS) overnight and then to 30% sucrose overnight till the brains sunk to the bottom of the tube. Serial sections of the brains were cut (30-μm sections) through each entire hippocampus using a freezing microtome. For immunofluorescence staining, after blocking with 10% bovine serum albumin (BAS) for 1 h at 20 ± 2 °C, the sections were incubated with the following primary antibodies at 4 °C overnight: anti-GFAP (MAB360, Millipore, 1:800 dilution), anti-NLRP3 (AG-20B-0014-C100, Adipogen, 1:400 dilution), anti-caspase-1 (AG-20B-0042, Adipogen, 1:400 dilution) and anti-GSDMD (ab219800, Abcam, 1:400 dilution). After that, the sections were incubated with Alexa Fluor 488-conjugated donkey anti-mouse IgG (Invitrogen, A21202; 1:1000 dilution), Alexa Fluor 488-conjugated goat anti-rabbit (Invitrogen, A11008; 1:1000 dilution), Alexa Fluor 555-conjugated goat anti-rabbit IgG (Invitrogen, 21432; 1:1000 dilution) or Alexa Fluor 555 goat anti-mouse IgG (Invitrogen, 21422; 1:1000 dilution) for 1 h at 20 °C. Then, the sections were washed and mounted onto glass slides. DAPI (P36931, Life Technologies) was used to visualize nuclei. Brain sections were imaged using a confocal microscope (Axiovert LSM510, Carl Zeiss Co., Germany). Images were then processed by ImageJ.

### Primary astrocyte culture and treatment

Astrocytes were prepared from the hippocampus of WT and CKO mice at P0-3, as described previously 5. The neonatal hippocampus tissue is trypsinized and dissociated. After centrifugation at 1000 g for 10 min, the cell pellets were resuspended in Dulbecco's modifed Eagle's medium/Ham's F12 medium containing 10% fetal bovine serum (FBS, GIBCO, Gaithersburg, MD, USA) and plated on poly-D-lysine (PDL)-coated T-75 flasks at 50,000 cells/cm^2^ to generate mixed glial cultures. Culture media was changed 24 h later to complete medium and subsequently twice a week. Confluent mixed glial cultures were shaken at 220 rpm for 6 h at 37 ◦C to remove unwanted cell types (microglia, oligodendrocytes, neurons, and fibroblasts). Astrocytes were released with 0.5% trypsin, and plated onto PDL-coated 6-well culture plates at density of 4 × 10^6^ cells. The purity of astrocytes was >95% as determined with GFAP immunocytochemistry. Astrocytes were primed with lipopolysaccharide (LPS, 100 ng ml^-1^, Sigma, USA) for 24 h and then pulsed with 5 mM ATP (Sigma, USA) for 30 min. For pharmacological measurements, NLRP3 inflammasome inhibitor VX765 (10 μM, Selleck, China) or ROS inhibitor N-acetyl-cysteine (NAC, 5 mM, Sigma, USA) was added to the cell culture medium 1 h before LPS plus ATP stimulation. The cell extracts and precipitated supernatants were analyzed by ELISA and immunoblotting.

### Elisa

The concentration of IL-1β in the cell culture supernatant and hippocampus tissues was measured by mouse IL-1β ELISA Kit (R&D, USA) according to the manufacturer's instructions. The level of lactate dehydrogenase (LDH) in culture medium was determined using a commercially available kit (Nanjing Jiancheng Bioengineering Institute, Nanjing, China) according to the manufacturer's instructions.

### Cell viability assay

Cell viability was measured by Cell Counting Kit-8 (CCK-8, Biotool, Houston, TX). Briefly, astrocytes isolated from the hippocampus of WT and CKO mice were plated on a 96-well plates at a density of 4 × 10^4^ cells per well and pretreated with VX765 for 1 h before LPS plus ATP stimulation. Next, 10 μl of CCK-8 reagent was added to each well for 4 h. Finally, the absorbance was measured at 450 nm using a multi-well spectrophotometer (Varioskan Flash, Thermo Fisher Scientific, USA). The cell viability of control cells was considered to be 100%.

### Measurement of mitochondrial superoxide

MitoSOX (M36008, Invitrogen, USA), mitochondrial superoxide indicator, is a novel fluorogenic dye for highly selective detection of superoxide in the mitochondria of live cells. Astrocytes were treated with LPS+ATP and then were stained with MitoSOX at 2.5 μM for 30 min at 37 °C. After that, the cells were washed with PBS twice and then resuspended in cold PBS containing 1% FBS for flow cytometric analyses. Flow data were analyzed with the FCS Express software (Guava Easy Cyte™8, Millipore, USA). For immunofluorescence, astrocytes were incubated with MitoSOX at 2.5 μM for 30 min at 37 °C and washed twice with PBS. The cells were then stained with DAPI for 10 min. Images were observed and photos were taken under a confocal microscope. Mitochondria-associated ROS levels were assessed by fluorescence intensity using Image J.

### Dye uptake

Dye uptake assay was performed as previously described 26. Astrocytes from the hippocampus of WT and CKO mice were incubated with VX765 1 h before LPS plus ATP treatment. YO-PRO-1 iodide (0.2 mM) and ethidium homodimer-2 (Eth-D2, 2 mM) were added to cell culture for 20 min at 37 °C. Cells were then stained with DAPI and images were captured using an inverted microscope (Olympus, Tokyo, Japan).

### Coimmunoprecipitation (Co-IP)

The total cell lysates were prepared from WT and CKO astrocytes and then incubated with anti-Kir6.1 (ab241996, Abcam, 1:500 dilution), anti-NLRP3 (AG-20B-0014-C100, Adipogen, 1:800 dilution) or anti-ASC (sc-271054, Santa Cruz Biotechnology, 1:1000 dilution) antibodies at 4 °C overnight, followed by an incubation with 20 μl protein A/G plus agarose (sc-2003, Santa Cruz Biotechnology, USA) for 4 h at 4 °C. After washing the beads, the bound proteins were eluted and analyzed by immunoblotting.

### Western blotting analysis

Cell lysates and tissues were homogenized in RIPA lysis buffer (Beyotime Biotechnology, China) and protein concentration was determined by the Bradford assay (Bio-Rad, Hercules, CA, USA). A 25-μg protein aliquot of each sample was separated and then electrophoretically transferred onto PVDF membranes (IPVH00010, Millipore, USA). Immuno-reactive bands were analyzed with ImageQuant™ LAS 4000 imaging system (GE Healthcare, Pittsburgh, PA, USA). Protein levels were determined by normalizing to the level of β-actin. The following primary and secondary antibodies were used: anti-NLRP3 (AG-20B-0014-C100, Adipogen), anti-caspase-1 (AG-20B-0042, Adipogen), anti-IL-1β (ab10626, Abcam), anti-GSDMD (ab219800, Abcam), anti-Kir6.1 (ab241996, Abcam), and anti-β-actin (BM0627, Boster, Pleasanton, CA, USA), Anti-mouse IgG, HRP-linked Antibody (7076, Cell Signaling Technology), Anti-rabbit IgG, HRP-linked Antibody (7074, Cell Signaling Technology).

### Statistical analysis

All data are expressed as means ± SEM. The data were collected and analyzed using GraphPad Prism 7 in a blinded manner. The differences with different treatments and genotypes were determined by one way or two-way analysis of variance (ANOVA), followed by the Tukey's post hoc test or Student's t-test and were considered as statistically significant at *p* < 0.05.

## Results

### The Kir6.1 expression is decreased in hippocampal astrocytes in mouse model of depression

**Figure 1:**
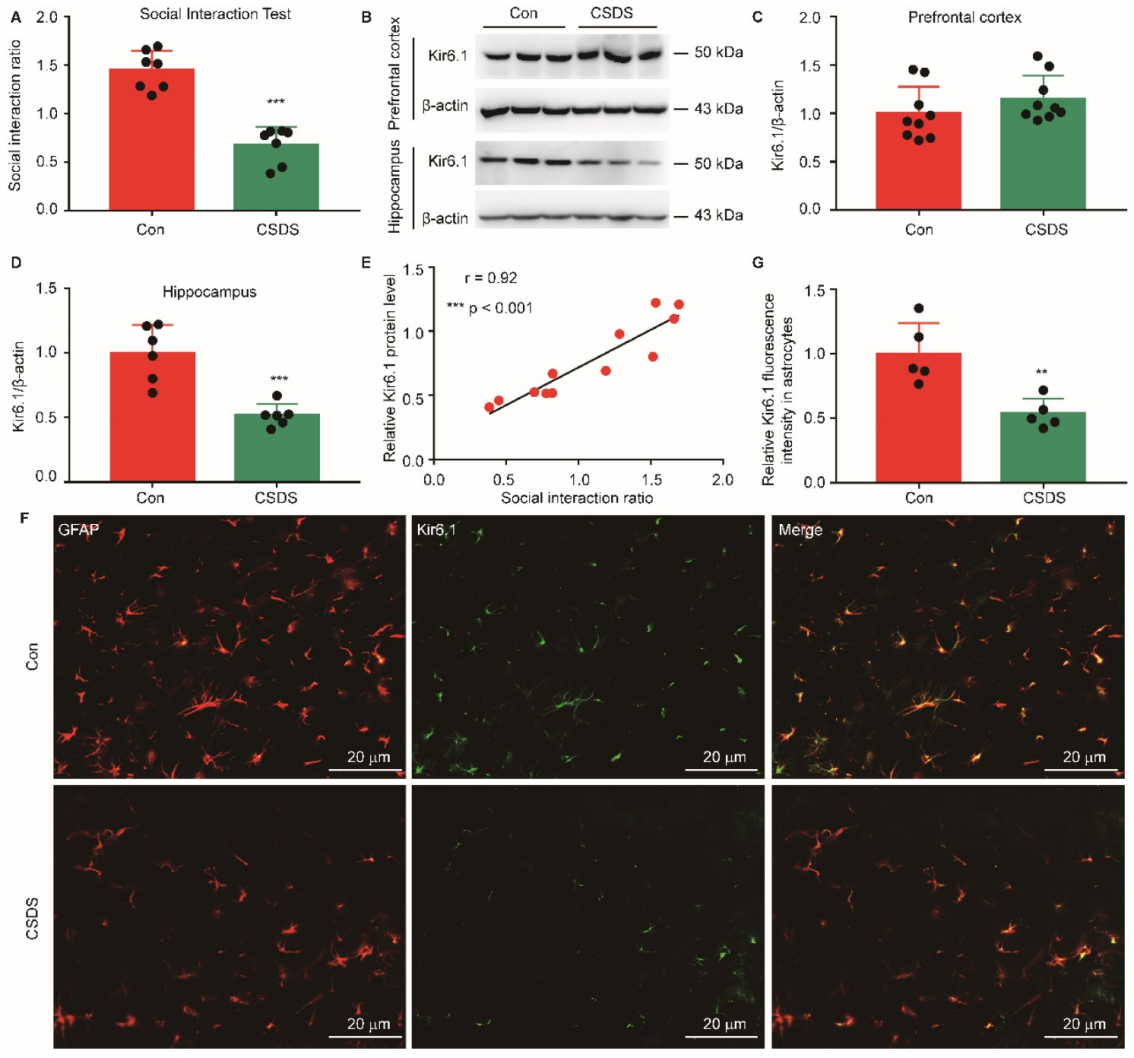
** Chronic stress reduced astrocytic Kir6.1 expression in the hippocampus. A** Social interaction ratio of CSDS and control (Con) mice (*n =* 7). **B-D** Representative immunoblots (B) and quantification of relative the Kir6.1 expression in the prefrontal cortex (C, *n* = 9) and hippocampus (D, *n* = 6) of mice after the CSDS. **E** The Kir6.1 protein in the hippocampus correlated with social avoidance (*n =* 6). **F-G** Representative images and quantification of the level of Kir6.1 (green) and GFAP (red) in the hippocampus of mice after the CSDS (*n =* 5). The data shown are the mean ± SEM. ^**^p < 0.01, ^***^p < 0.001. Two-tailed unpaired t-test (A, C, D, G) and correlation was analyzed by Pearson's correlation coefficient (E).

To investigate the aberrant expression of Kir6.1 in depression, we established CSDS-induced mouse model of depression. CSDS caused a social avoidance behavior as measured by the social interaction (**Figure 1A, *p <* 0.001**). The Kir6.1 protein level in the hippocampus and medial prefrontal cortex, which are strongly relevant to depression, was detected by western blot analysis. We found that the Kir6.1 expression was markedly reduced in the hippocampus after CSDS (***p <* 0.001**), not in medial prefrontal cortex **(*p =* 0.2323)** (**Figure 1B-D**). Additionally, the level of Kir6.1 was positively correlated with the social interaction ratio in mouse model of depression (**Figure 1E,** r = **0.92, *p <* 0.001**). Kir6.1 is mainly expressed in astrocytes in the brain. To determine the level of astrocytic Kir6.1 under stress, the hippocampus slices of mice were stained to visualize Kir6.1 with GFAP (a marker of astrocytes). Immunostaining and quantification revealed that the Kir6.1 protein level was markedly decreased in astrocytes in the hippocampus after CSDS (**Figure 1F-G, *p =* 0.0044**). These results indicate that astrocytic Kir6.1 expression is reduced in the hippocampus of mouse model of depression.

### Astrocyte-specific knockout of Kir6.1 exacerbates CUMS-induced depressive-like behaviors in mice

**Figure 2:**
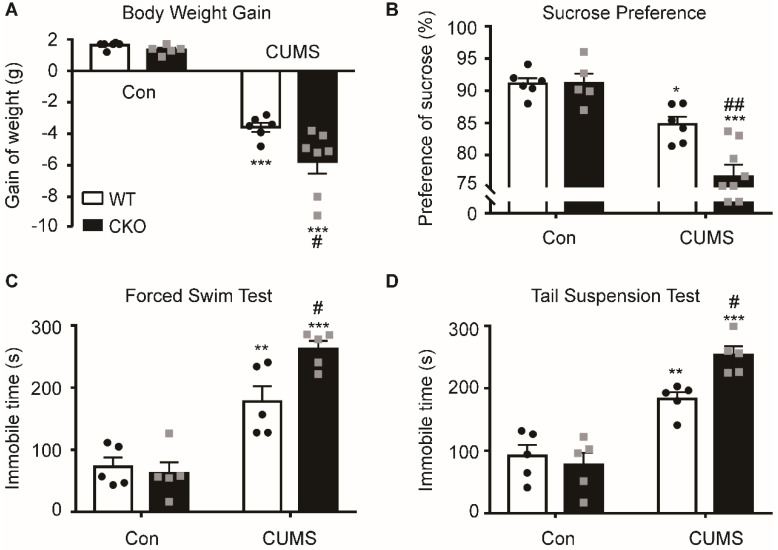
** Astrocyte-specific knockout of Kir6.1 exacerbates CUMS-induced depressive-like behaviors in mice. A-D** Body weight gain (A), sucrose preference (B), total immobility time in the FST (C) and in TST (D) of WT and CKO mice following 5 weeks of CUMS (*n =* 5-8). The data shown are the mean ± SEM. ^*^p < 0.05, ^**^p < 0.01, ^***^p < 0.001 vs corresponding control (Con) group; ^#^*p <* 0.05, ^##^*p <* 0.01 vs WT CUMS groups.

To study the role of astrocytic Kir6.1/K-ATP channel in depression, astrocytic Kir6.1 CKO mice were subjected to the CUMS procedure and their depressive behaviors were analyzed. CKO mice exhibited a significant decrease in body weight increase **(*p =* 0.0171)** and in the sucrose preference **(*p =* 0.0035)** compared to WT mice after the CUMS paradigm (**Figure 2A-B**). In addition, the duration of immobility in TST **(*p =* 0.0264)** and FST **(*p =* 0.0217)** was significantly longer in CKO mice than in WT mice after the CUMS procedure (**Figure 2C-D**). These data demonstrate that astrocytic Kir6.1 deletion induces depressive behaviors in adult mice.

### Astrocytic Kir6.1 ablation aggravates CSDS-induced depressive-like behaviors in mice

**Figure 3:**
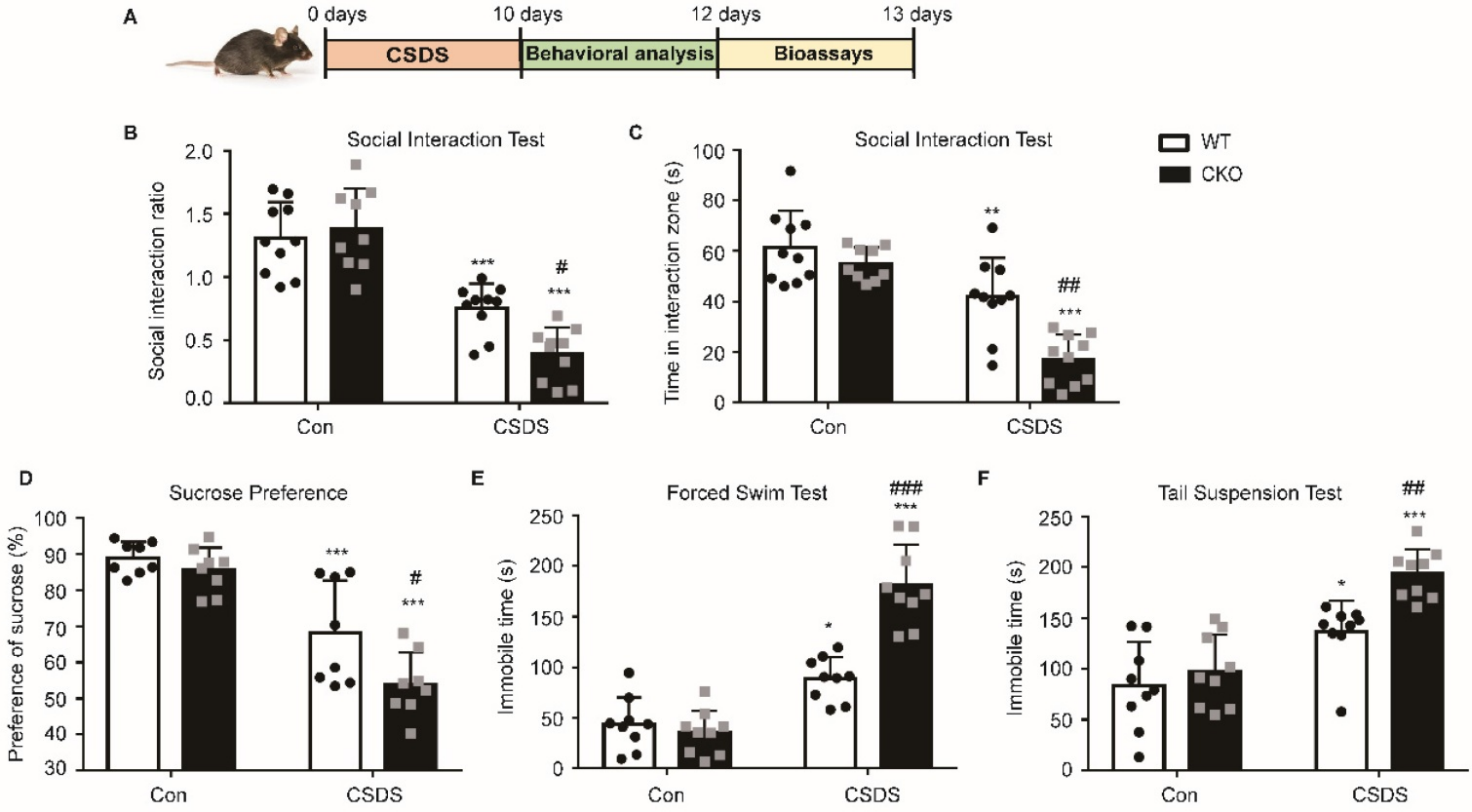
** Astrocytic Kir6.1 ablation aggravates CSDS-induced depressive-like behaviors in mice. A** CSDS paradigm.** B** Social avoidance behaviors of WT and CKO mice after CSDS. **C** Time spent in the interaction zone with novel target. **D** Sucrose preference of mice after CSDS. **E-F** Total immobility time in FST (E) and in TST (F) of mice after CSDS. The data shown are the mean ± SEM, *n =* 8-10. ^*^p < 0.05, ^**^p < 0.01, ^***^p < 0.001 vs corresponding control (Con) group; ^#^*p <* 0.05, ^##^*p <* 0.01, ^###^*p <* 0.001 vs WT CSDS groups.

To further confirm the role of astrocytic Kir6.1/K-ATP channel in depression, CKO mice and WT mice were subjected to the CSDS procedure and their depressive behaviors were analyzed **(Figure 3A)**. Compared to WT mice, CKO mice displayed a lower social interaction ratio **(*p =* 0.0165)** and less time spent in the interaction zone when the social target was present **(*p <* 0.01)** (**Figure 3B-C**). Importantly, a smaller percentage of CKO were resilient to CSDS (defined as social interaction ratio > 1, 47% vs 26%). In addition, CKO mice showed a significant decrease in the sucrose preference and a significant increase **(*p =* 0.0220)** in duration of immobility in FST **(*p <* 0.001)** and TST **(*p =* 0.0066)** compared to WT mice after the CSDS paradigm **(Figure 3D-F)**. These data indicate that astrocytic Kir6.1 deletion aggravates depression-like behaviors in mice.

### Astrocytic Kir6.1 deficiency enhances astrocyte injury and NLRP3-related pyroptosis in the hippocampus

**Figure 4:**
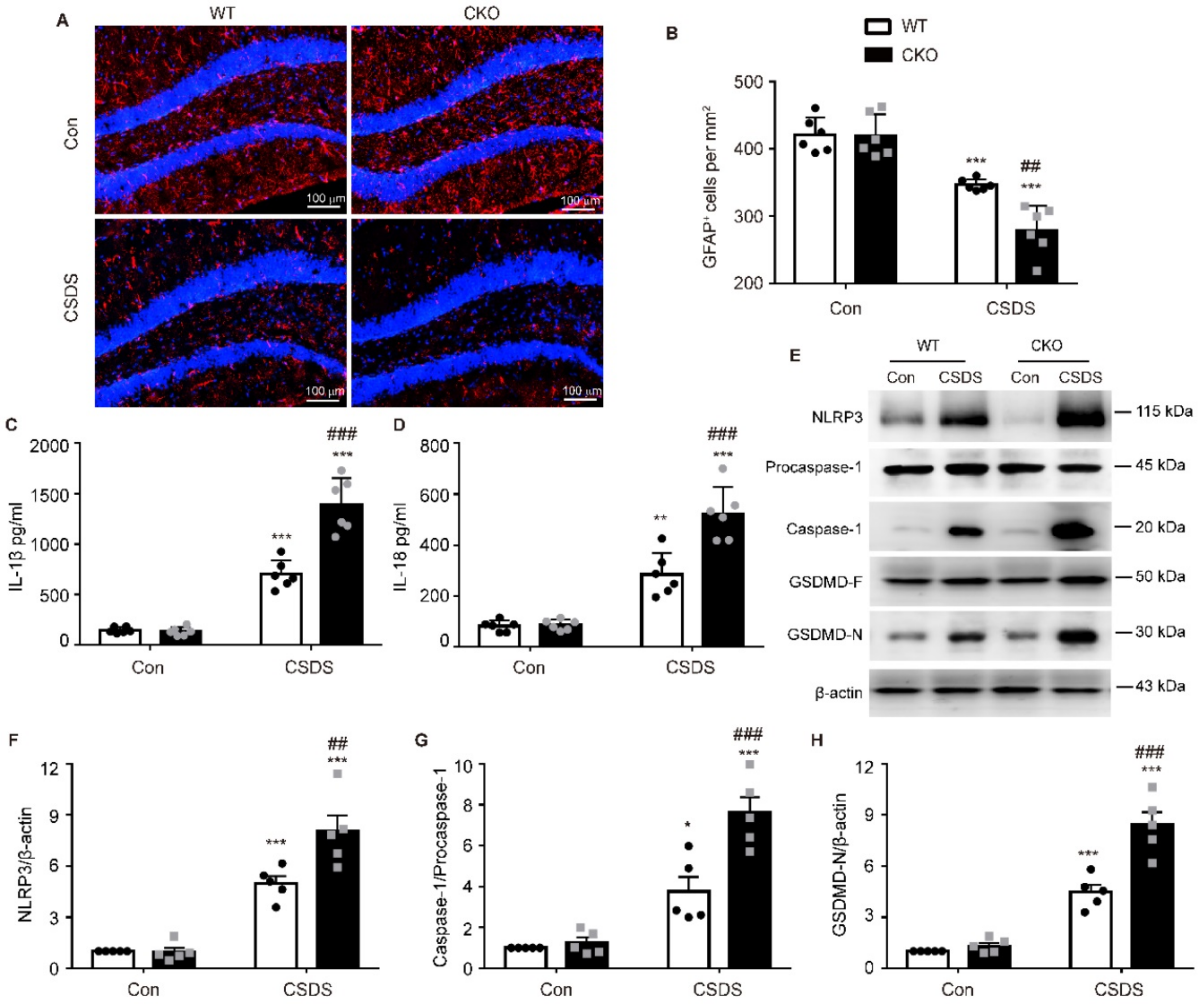
** Astrocytic Kir6.1 deletion aggravates astrocyte injury and NLRP3-related pyroptosis in the hippocampus. A-B** Immunofluorescent staining of GFAP-positive astrocytes in hippocampal sections (A) with quantification (B, *n =* 6). **C-D** ELISA of IL-1β (C) and IL-18 (D) from hippocampus of WT and CKO mice after CSDS (*n =* 6). **E** Representative immunoblots of NLRP3, caspase-1, and GSDMD-N from mice hippocampus. **F-H** Quantification of NLRP3 (F), caspase-1 (G), and GSDMD-N (H) (*n =* 5). The data shown are the mean ± SEM. ^*^p < 0.05, ^**^p < 0.01, ^***^p < 0.001 vs corresponding control (Con) group; ^##^*p <* 0.01, ^###^*p <* 0.001 vs WT CSDS groups.

Astrocytes play a crucial role in the pathogenesis of depression 27. Therefore, we then investigated the number of astrocytes by detecting their marker GFAP. As shown in **Figure 4A-B**, compared with WT mice, the number of GFAP-positive cells in the hippocampus of CKO mice was significantly reduced after CSDS **(*p =* 0.0021)**, indicating that astrocytic Kir6.1 deficiency promotes the loss of astrocytes in depression. Increasing evidence has demonstrated that pyroptosis is associated with astrocytes loss in depression 28. So we detected the pyroptosis-related protein expression in the hippocampus. CKO mice displayed higher levels of NLRP3 **(*p =* 0.0054)**, caspase-1 cleavage **(*p =* 0.0007)**, GSDMD-N **(*p <* 0.001)**, IL-1β **(*p <* 0.001)** and IL-18 **(*p <* 0.001)** following CSDS treatment than WT mice (**Figure 4C-H**). These results demonstrate that astrocytic Kir6.1 deficiency enhances astrocyte loss and NLRP3-related pyroptosis in the hippocampus.

### Astrocytic Kir6.1 knockout increases NLRP3-mediated pyroptosis in astrocytes

**Figure 5:**
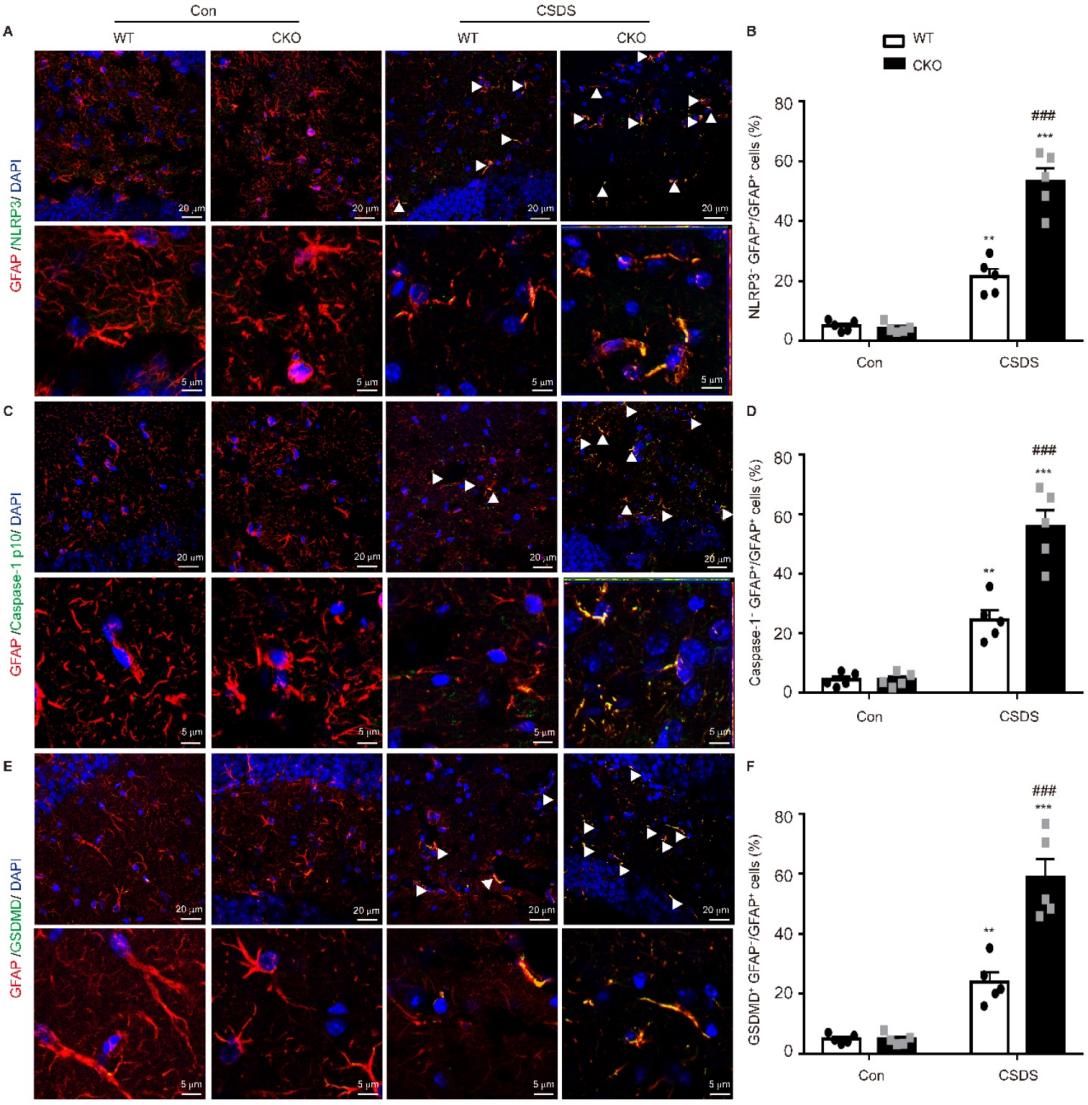
** Astrocytic Kir6.1 ablation promotes pyroptosis of astrocytes in the hippocampus. A, C, E** Representative magnification images showing the co-localization of GFAP (red) and NLRP3 (green) (A), GFAP (red) and caspase-1 (green) (C), and GFAP (red) and GSDMD (green) (E) in a part of mice hippocampus region after CSDS. White arrows show example of GFAP^+^/NLRP3^+^, GFAP^+^/caspase-1^+^, and GFAP^+^/GSDMD^+^ cells. **B, D, F** Quantification of the percentage of GFAP positive cells that are NLRP3 positive (B), caspase-1 positive (D), and GSDMD positive (F) in the hippocampus (*n =* 5). The data shown are the mean ± SEM. ^**^p < 0.01, ^***^p < 0.001 vs corresponding control (Con) group; ^###^*p <* 0.001 vs WT CSDS groups.

Subsequently, we wanted to determine in which cell type NLRP3-mediated pyroptosis occurs predominantly. Double immunostaining analysis showed that NLRP3-related protein strongly co-localized with the astrocytic marker GFAP, indicating NLRP3-mediated pyroptosis mainly occurs in astrocytes **(Figure 5, Figure S1)**. More importantly, the levels of NLRP3 **(*p <* 0.001)**, caspase-1 **(*p <* 0.001)** and GSDMD **(*p <* 0.001)** were substantially higher in GFAP-positive astrocytes in CKO mice than in WT mice **(Figure 5)**. These findings illustrate that astrocytic Kir6.1 ablation promotes NLRP3-mediated astrocytic pyroptosis in the hippocampus of mouse model of depression.

### Kir6.1 is an essential negative regulator of NLRP3-mediated astrocytic pyroptosis

**Figure 6:**
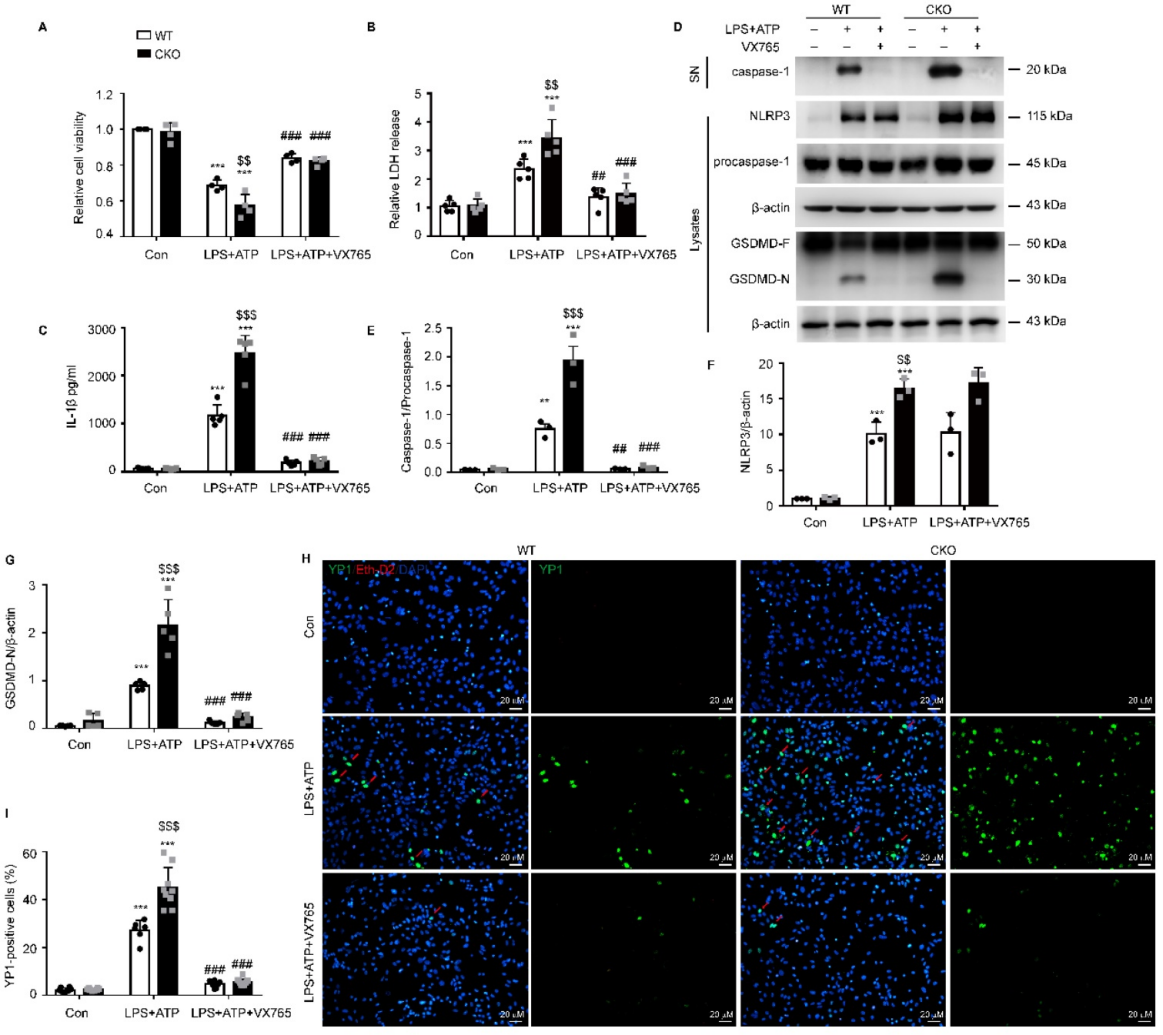
** Kir6.1 is a negative regulator of NLRP3-mediated astrocytic pyroptosis.** Primary astrocytes prepared from WT and CKO mice were treated with 10 µM VX765 for 1 h, followed by stimulation with LPS plus ATP. **A** The viability of cells was assessed by the CCK-8 assay (*n =* 4). **B** LDH in supernatants was measured by LDH kit (*n =* 5). **C** ELISA of IL-1β in the supernatants (*n =* 5). **D-G** Representative immunoblots of the cleaved caspase-1 in the supernatants (SN) and the NLRP3, pro-caspase-1, full-length GSDMD and GSDMD N-terminal in cell lysate (D) and quantification of cleaved caspase-1 (E, *n =* 3), NLRP3 (F, *n =* 3) and GSDMD-N (G, *n =* 5). **H** Treated astrocytes were stained with YO-PRO-1 (green) and Eth-D2 (red) to visualize the discrete membrane pores. DAPI stains nucleus (blue). **I** Quantification of YO-PRO-1^+^ Eth-D2^-^ cells (*n =* 5). The data shown are the mean ± SEM. ^**^p < 0.01, ^***^p < 0.001 vs corresponding control (Con) group; ^$$^*p <* 0.01, ^$$$^*p <* 0.001 vs WT LPS+ATP groups; ^##^*p <* 0.01, ^###^*p <* 0.001 vs corresponding LPS+ATP group.

To further study the regulation of NLRP3-mediated pyroptosis by Kir6.1, astrocytes were isolated from WT and CKO mice and treated with the NLRP3 inflammasome inhibitor VX765. We found that astrocyte injury **(*p =* 0.0060)** and LDH release through the pyroptotic pores **(*p =* 0.0020)** were significantly enhanced in CKO astrocytes and these enhancements were reversed by VX765 treatment (**Figure 6A-B, *p <* 0.001**). In addition, the NLRP3 expression **(*p =* 0.0067)**, caspase-1 activation **(*p <* 0.001)**, and IL-1β maturation **(*p <* 0.001)** were significantly increased in CKO astrocytes after LPS and ATP stimulation (**Figure 6 C-F**). More importantly, the level of the GSDMD N-terminus was notably higher in astrocytes from CKO mice than WT mice (**Figure 6G, *p <* 0.001**). Additionally, VX765 could reverse the enhanced caspase-1 activation, IL-1β secretion, and GSDMD N-terminus cleavage in CKO astrocytes** (*p <* 0.001)**. The formation of pores is a unique feature of pyroptosis. The pores allows only small molecules to pass through. Thus, the combined use of Eth-D2, a larger membrane impermeable dye, and YO-PRO-1 iodide, a small membrane impermeable dye, allows visualization of pyroptosis-related pores 29. We found that the uptake of YO-PRO-1 iodide (green) in Kir6.1 KO astrocytes **(*p <* 0.001)** was notably increased and this increase was also reversed by VX765 pretreatment (**Figure 6H-I, *p <* 0.001**). These data indicate that Kir6.1 negatively regulates NLRP3-mediated pyroptosis in astrocytes.

### Kir6.1 prevents NLRP3 inflammasome assembly via interaction with NLRP3

**Figure 7:**
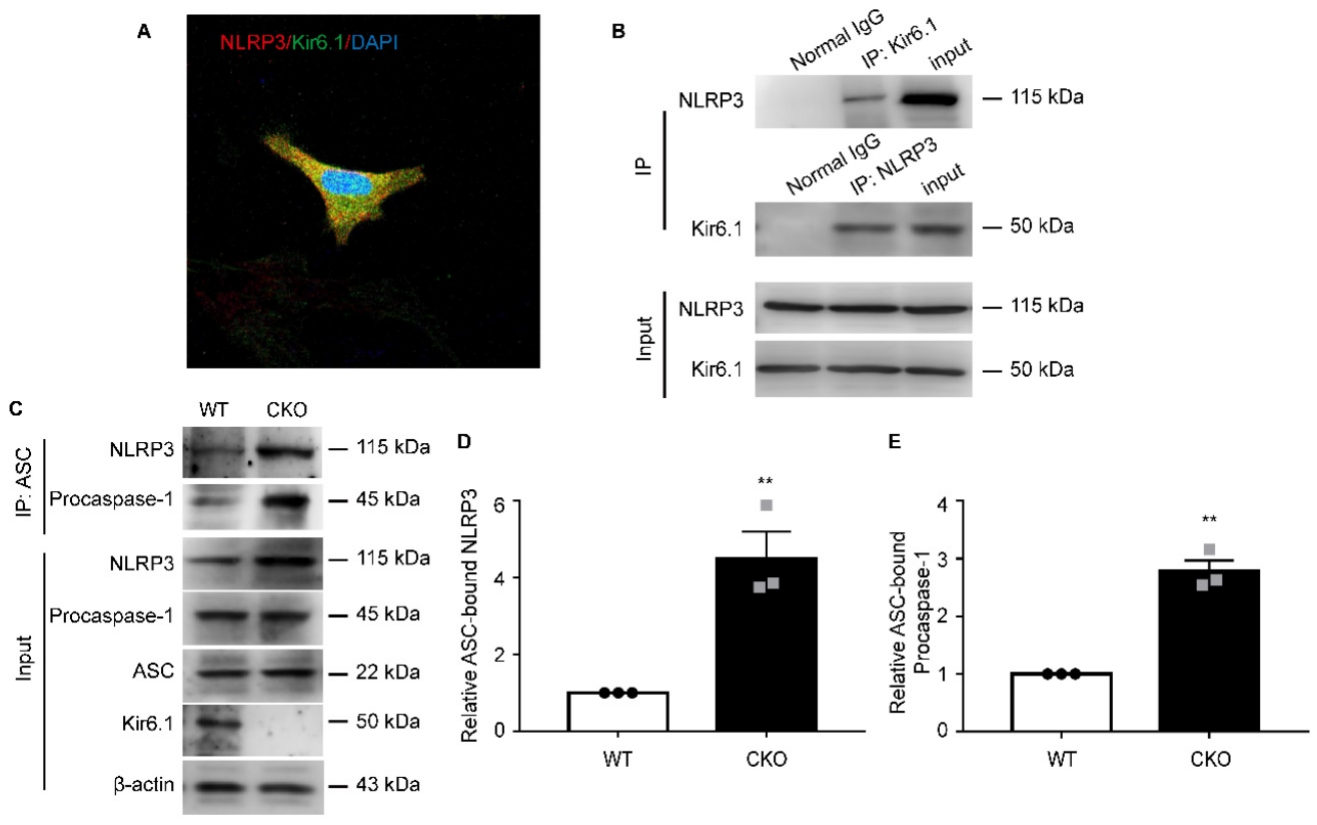
** Kir6.1 interacts with NLRP3 and inhibits NLRP3 inflammasome assembly. A** Representative images showing the localization of NLRP3 (red) and Kir6.1 (green) in astrocytes. **B** The interaction between Kir 6.1 and NLRP3 in astrocytes was measured by co-IP. **C** The association of ASC with NLRP3, and procaspase-1 in LPS+ATP treated astrocytes isolated from WT and CKO mice was assessed by co-IP. **D-E** Quantification of data shown in (C, *n =* 3). The data shown are the mean ± SEM. ^**^p < 0.01.

To dissect the underlying molecular mechanisms, we first examined whether there is an interaction between Kir6.1 and NLRP3 in astrocytes. co-IP assays showed that Kir6.1 successfully interacted with NLRP3 in astrocytes. The association of Kir6.1 with NLRP3 was further confirmed by immunofluorescence co-localization in astrocytes (**Figure 7A-B**). The assembly of NLRP3 inflammasome subunits (NLRP3, ASC, and procaspase-1) is a prerequisite for its activation. We then wondered whether this interaction of Kir6.1 with NLRP3 interferes with the assembly of NLRP3 imflammasome subunits. Indeed, the amounts of ASC-bound NLRP3 and procaspase-1 in the immunoprecipitates were significantly higher in CKO astrocytes than WT astrocytes (**Figure 7C-E, *p =* 0.0073**). These results suggest that Kir6.1 prevents the NLRP3 inflammasome assembly through association with NLRP3 in astrocytes.

### Kir6.1 deletion-induced mitochondrial ROS is required for astrocytic pyroptosis

**Figure 8:**
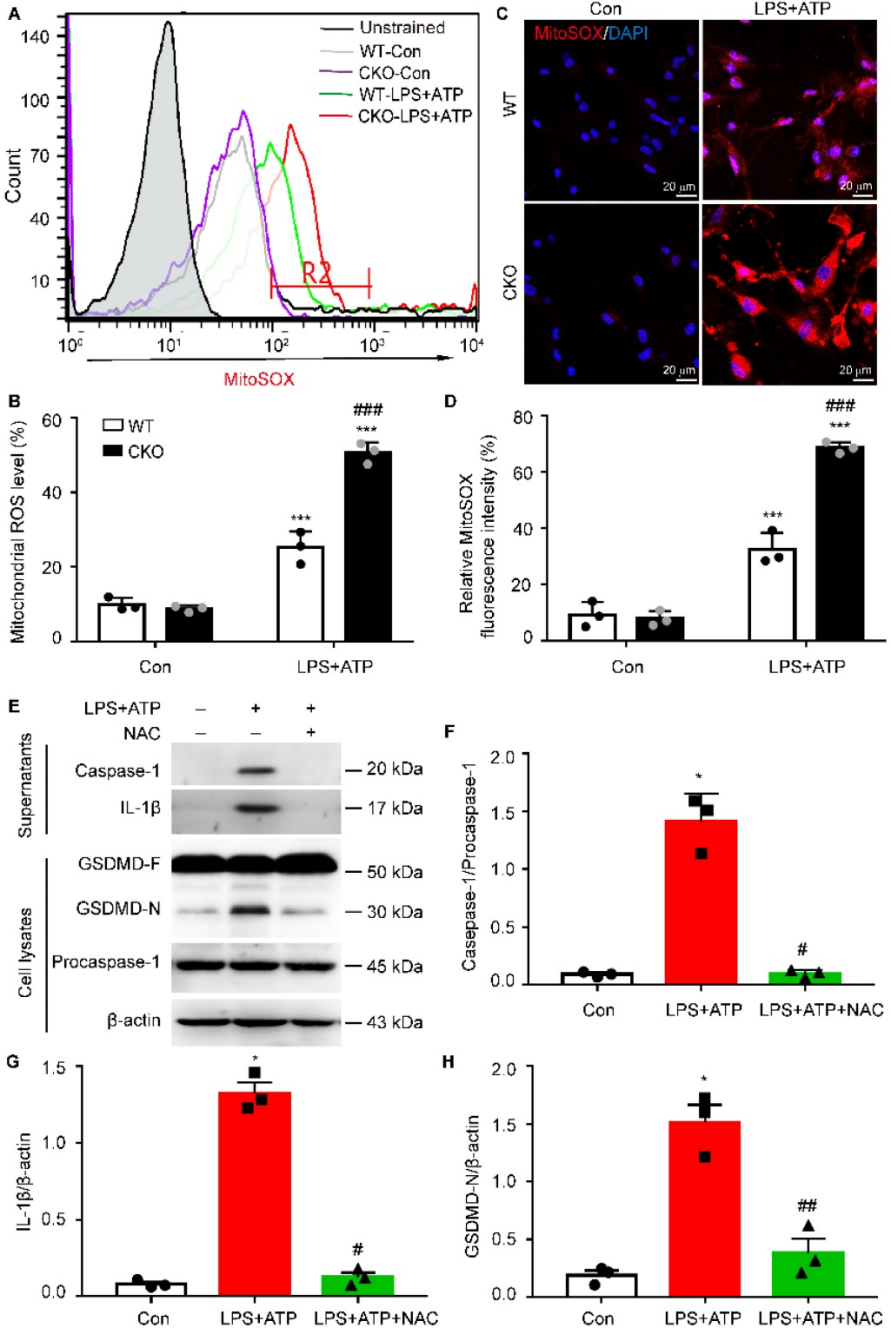
** The mitochondrial ROS is required for NLRP3-mediated pyroptosis in Kir6.1 knockout astrocytes. A-B** Representative images and quantification of mitochondrial ROS levels by flow cytometry (*n =* 3). **C-D** Representative images and quantification of MitoSOX fluorescence intensity (*n =* 3). The nucleus was stained with DAPI (blue). ^***^p < 0.001 vs control (Con) group; ^###^*p <* 0.001 vs WT LPS+ATP groups. Primary astrocytes from CKO mice were pretreated with 5 mM ROS inhibitor NAC for 1 h and then stimulated with LPS plus ATP. **E-H** Representative immunoblots of the cleaved caspase-1 and mature IL-1β in the supernatants and the GSDMD and GSDMD N-terminal in cell lysates (E) and quantification of caspase-1 cleavage (F), IL-1β (G), and GSDMD-N (H). *n =* 3, ^*^p < 0.05 vs control (Con) group; ^#^*p <* 0.05, ^##^*p <* 0.01 vs LPS+ATP groups.

Increasing evidence suggests that mitochondrial dysfunction is associated with NLRP3 inflammasome activation 30, 31. Therefore, we then examined the generation of mitochondrial ROS (mtROS) in CKO astrocytes and WT controls. As shown in **Figure 8A-B**, the robust ROS-generating mitochondria was significantly higher, as determined by MitoSOX staining, in CKO astrocytes than WT controls **(*p <* 0.001)**. This increased mtROS production in CKO astrocytes were further confirmed by the fluorescence of MitoSOX (**Figure 8C-D, *p <* 0.001**). To determine whether Kir6.1 knockout-induced mtROS contributes to NLRP3-mediated astrocytic pyroptosis, the CKO astrocytes were pretreated with the ROS inhibitor NAC. The NAC effectively reversed the increase in the level of the GSDMD N-terminus **(*p =* 0.0094)**, IL-1β secretion **(*p =* 0.0123)** and caspase-1 activation** (*p =* 0.0227)** (**Figure 8E-H**). These results indicate that Kir6.1 deletion enhances astrocytic pyroptosis through the mtROS-NLRP3-GSDMD signaling in astrocytes.

### Inhibition of NLRP3 inflammasome rescues astrocytic pyroptosis and depressive behaviors in CKO mice

**Figure 9:**
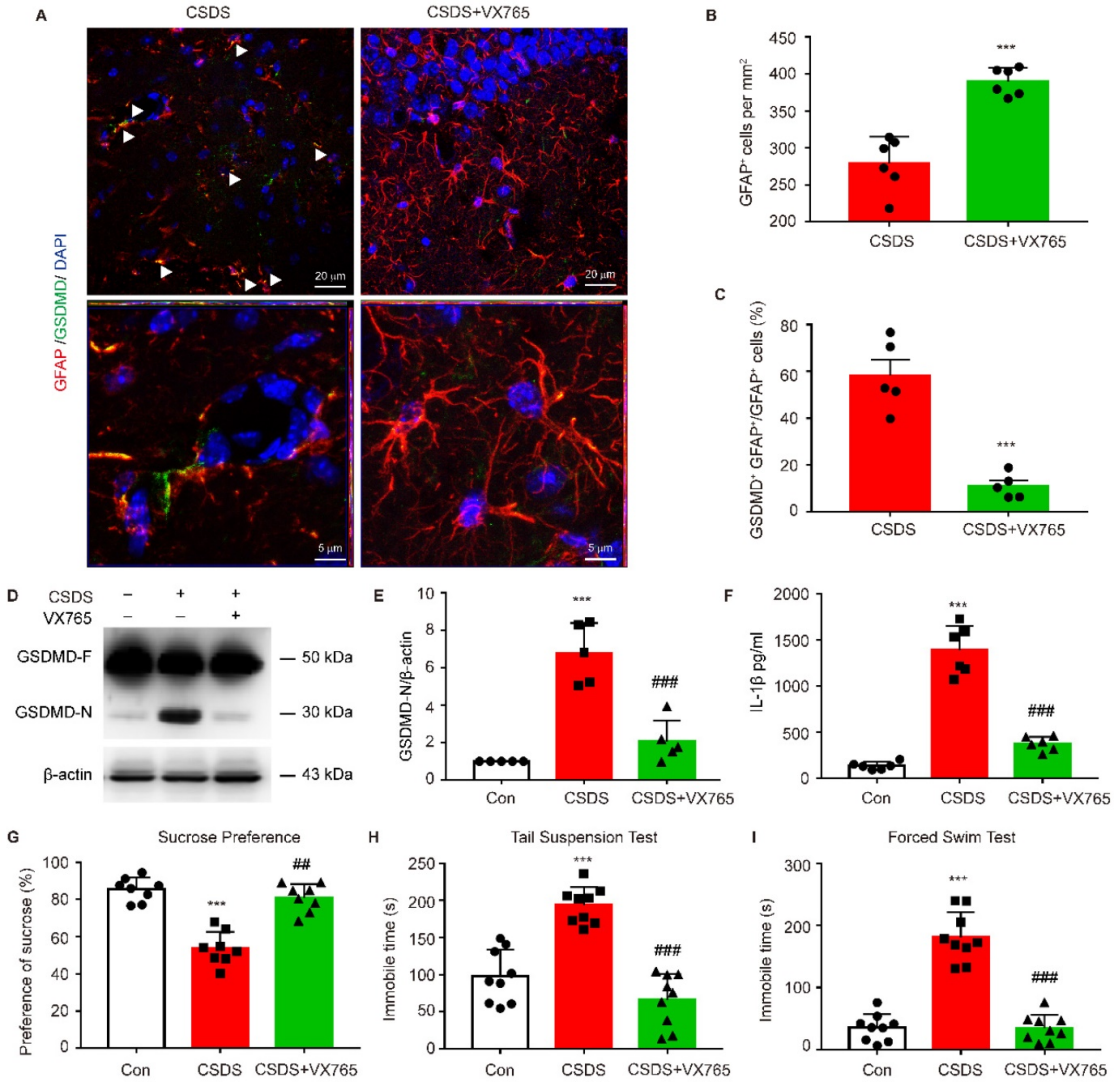
** Inhibiting NLRP3 inflammasome rescues pyroptosis of astrocytes and depressive behaviors in CKO mice.** CKO mice were intraperitoneally injected with VX765 (100 mg/kg) once daily for 10 consecutive days. **A** Representative magnification images showing the co-localization of GFAP (red) and GSDMD (green) in a part of mice hippocampus region after CSDS. White arrows show example of GFAP^+^/GSDMD^+^ cells. **B-C** Quantification of GFAP^+^ cells number (B, *n =* 6) and percentage of GFAP positive cells that are GSDMD positive (C, *n =* 5) in the DG area of hippocampus. ^**^p < 0.01, ^***^p < 0.001. **D-E** Representative immunoblot (D) and quantitative analysis of GSDMD in hippocampus of mice (E, *n =* 5). **F** ELISA of IL-1β from hippocampus of mice (*n =* 6). **G-I** Sucrose preference (G, *n =* 8), total immobility time in TST (H, *n =* 9) and in FST (I, *n =* 9) of mice. ^***^p < 0.001 vs control (Con) group; ^##^*p <* 0.01, ^###^*p <* 0.001 vs CSDS groups.

To further determine whether astrocytic pyroptosis and depressive behaviors observed in CKO mice was indeed caused by enhanced NLRP3 inflammasome activation, CKO mice were treated with NLRP3 inflammasome inhibitor VX765. VX765 treatment markedly increased the number of astrocytes and significantly decreased the number of GFAP^+^ GSDMD^+^ cells in hippocampus (**Figure 9A-C, *p <* 0.001**). VX765 also reduced the GSDMD N-terminus expression and IL-1β secretion in hippocampus (**Figure 9D-F, *p <* 0.001**). Moreover, this treatment significantly rescued depressive-like behaviors in CKO mice including a considerable increase in the sucrose preference **(*p =* 0.0020)** and a significant decrease in the duration of immobility in TST and FST (**Figure 9G-I, *p <* 0.001**). These findings demonstrate that astrocytic Kir6.1 ablation exacerbates depressive-like behaviors via enhancing NLRP3-mediated astrocytic pyroptosis.

## Discussion

In this study, we found that chronic stress reduced astrocytic Kir6.1 expression in the hippocampus. Furthermore, astrocyte-specific knockout of Kir6.1 aggravated depressive-like behaviors in mice. Astrocytic Kir6.1 deletion increased pyroptosis of astrocytes via the mtROS-NLRP3-GSDMD signaling pathway.

The most important finding presented here is that Kir6.1/K-ATP channel in astrocytes plays a crucial role in the pathogenesis of depression. As a metabolic gatekeeper, the K-ATP channel is extensively distributed in brain neurons and glial cells, specifically located at the membranes of cell and mitochondrial organelle 19, 22. Our previous studies showed that K-ATP channel opener could improve depressive behavior via inhibition of inflammation in mouse hypothalamus 32, while Kir6.2 knockout aggravated depressive behavior by promoting CA3 neuron death 21. However, the exact effect of Kir6.1/K-ATP channel in depression is still unknown. Here, we observed that chronic stress reduced astrocytic Kir6.1 expression in the hippocampus. We then used astrocyte-specific knockout of Kir6.1 mice to prepare two well-known mouse models of depression (CUMS and CSDS) and found that Kir6.1 CKO mice exhibited more severe depressive-like behavior including the lower social interaction ratio and sucrose preference and the longer the duration of immobility in FST and TST. Moreover, we also found that deleterious behaviors observed in Kir6.1 CKO mice were accompanied with enhanced NLRP3-mediated astrocytic pyroptosis. Furthermore, treatment with NLRP3 inflammasome inhibitor VX765 almost reversed the astrocytic pyroptosis and rescued the depressive behaviors in CKO mice. These findings indicate that astrocytic Kir6.1 ablation exacerbates depressive-like behaviors, at least partially, via promoting NLRP3-mediated astrocytic pyroptosis in mice.

Another important finding described here is that Kir6.1 is an essential negative modulator of pyroptosis in astrocytes. Pyroptosis is GSDMD-mediated programmed cell death 33. Recent studies have shown that pyroptosis plays a vital role in the death process of neurons 34, 35 and is implicated in the pathophysiology of many central nervous system diseases including depression 36, 37. Therefore, the functional manipulation of the pyroptosis has been thought to be a promising therapeutic strategy for these diseases. Here, we showed that astrocytic Kir6.1 deficiency enhanced the pyroptosis-associated proteins expression including NLRP3, caspase-1 activation, GSDMD-N cleavage and IL-1β and LDH release in primary astrocytes and in the hippocampus of mice as evidenced by Western blot and ELISA. In addition to GSDMD cleavage, pore formation on the plasma membrane is another functional characterization of pyroptosis, which allows only molecules with a diameter less than 10-13 nm to pass through. Thus, small (YO-PRO-1 iodide, 629 Da) pore permeable and large (Eth-D2, 1,293 Da) pore impermeable dyes were combined to visualize pyroptotic cells by fluorescence microscopy. This dye uptake assay further confirmed that deletion of Kir6.1 in astrocytes significantly promoted the pyroptotic pore formation on the plasma membrane in astrocytes. These findings demonstrate that Kir6.1 is an important negative regulator of astrocytic pyroptosis.

**Figure 10:**
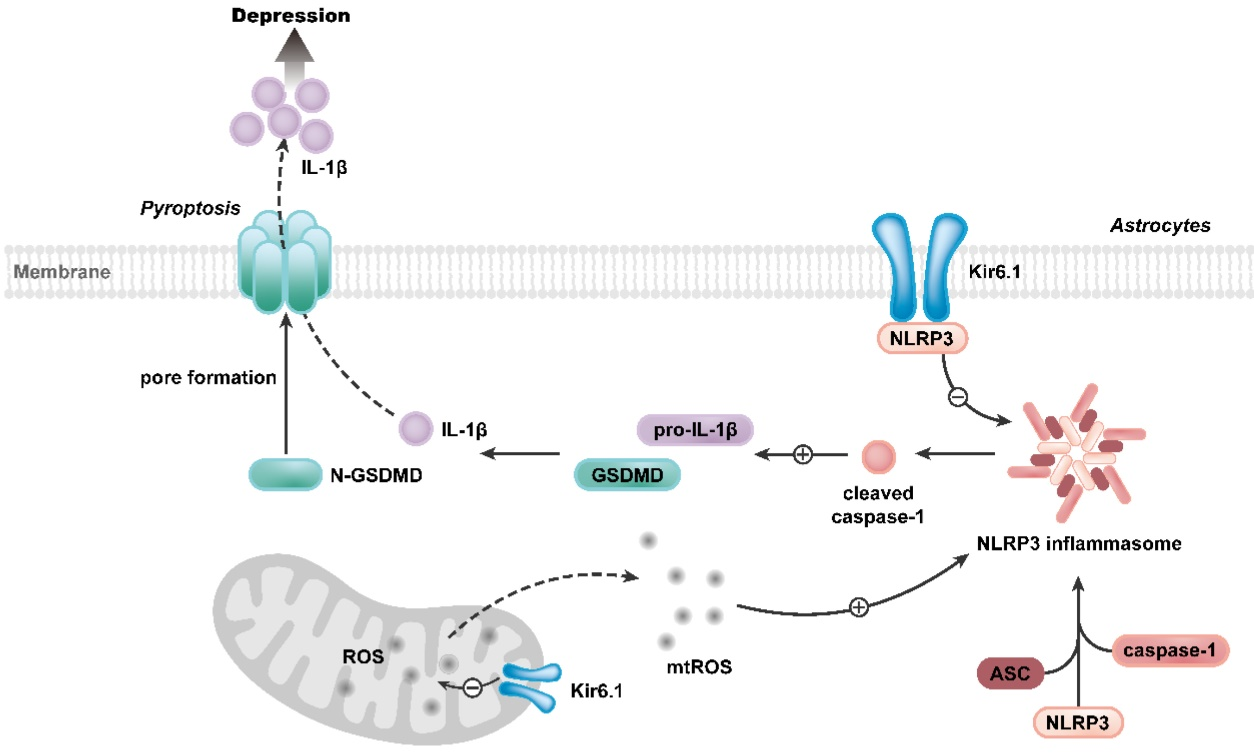
Proposed model depicting the crucial role of Kir6.1, via its interaction with NLRP3 and inhibition of mtROS generation, in preventing the assembly and activation of NLRP3 inflammasome, consequently, inhibiting the pyroptosis of astrocytes in depression.

Our study further reveals the molecular mechanism underlying the function of Kir6.1 in regulating astrocytic pyroptosis. The NLRP3 inflammasome is highly expressed in astrocytes and especially relevant to pyroptosis 38, 39. In the present study, we found that the expression of NLRP3 were significantly higher in CKO mice than WT mice after the exposure to CSDS. We also observed that, compared with WT mice, CKO mice displayed a significant increase in the activation of caspase-1, GSDMD N-terminus cleavage and the maturation of IL-1β following CSDS. The similar results were further observed in astrocytes from CKO mice. Furthermore, NLRP3 inflammasome inhibitor VX765 almost abolished the astrocytic pyroptosis in CKO astrocytes, indicating that Kir6.1 deletion-induced astrocytic pyroptosis is dependent on NLRP3 inflammasome. The assembly of NLRP3 inflammasome is a prerequisite for its activation 40-42. Here, we found that Kir6.1 could bind to NLRP3 and, in absence of Kir6.1, the interaction between NLRP3 inflammasome subunits was significantly enhanced in astrocytes. The possible explanation is that the interaction of Kir6.1 with NLRP3 interferes with the binding of NLRP3 to ASC and procaspase-1 subunits, thereby preventing the assembly and activation of NLRP3 inflammasome. The production of mitochondrial ROS is believed to be a common activator of the NLRP3 inflammasome 43, 44. Kir6.1/K-ATP channels are located in the mitochondria and normalize mitochondrial function 45. Here, we found that astrocytic Kir6.1 knockout significantly increased the mitochondrial ROS production. More importantly, ROS inhibitor NAC almost abolished the NLRP3-mediated astrocytic pyroptosis in Kir6.1 knockout astrocytes. Collectively, these findings indicate that Kir6.1 suppresses astrocytic pyroptosis by inhibiting the mtROS-NLRP3-GSDMD signal pathway in depression (**Figure 10**).

In conclusion, our findings demonstrate that Kir6.1/K-ATP channel in astrocytes is an essential negative regulator of astrocytic pyroptosis and plays a crucial role in depression. Our work suggests that Kir6.1/K-ATP channel may be a promising therapeutic target for depression.

## Supplementary Material

Supplementary figure.

## Abbreviations

K-ATP channels: ATP-sensitive potassium channels
Kir6.1/K-ATP channel: Kir6.1-contaning K-ATP channel
NLRP3: NOD-like receptor protein 3
GSDMD: Gasdermin D
ASC: apoptosis-associated speck-like protein
CUMS: chronic unpredictable mild stress
CSDS: chronic social defeat stress
GFAP: glial fibrillary acidic protein
CCK-8: Cell Counting Kit-8
LDH: Lactate dehydrogenase
ROS: reactive oxygen species
CKO: Kir6.1^loxp/loxp^ GFAP^Cre^
WT: Kir6.1^loxp/loxp^
TST: Tail suspension test
FST: Forced swim test
