# Supplementary material for: Kir6.1/K-ATP channel in astrocytes is an essential negative modulator of astrocytic pyroptosis in mouse model of depression
Source: Theranostics. 2022 Sep 11;12(15):6611–25. doi: 10.7150/thno.77455 (PMC9516231; doi:10.7150/thno.77455)
Supplement: Supplementary file 1 — Supplementary figure. [file thnov12p6611s1.pdf]

NLRP3-mediated pyroptosis protein was not mainly expressed in microglia in CSDS-induced mouse model of depression.

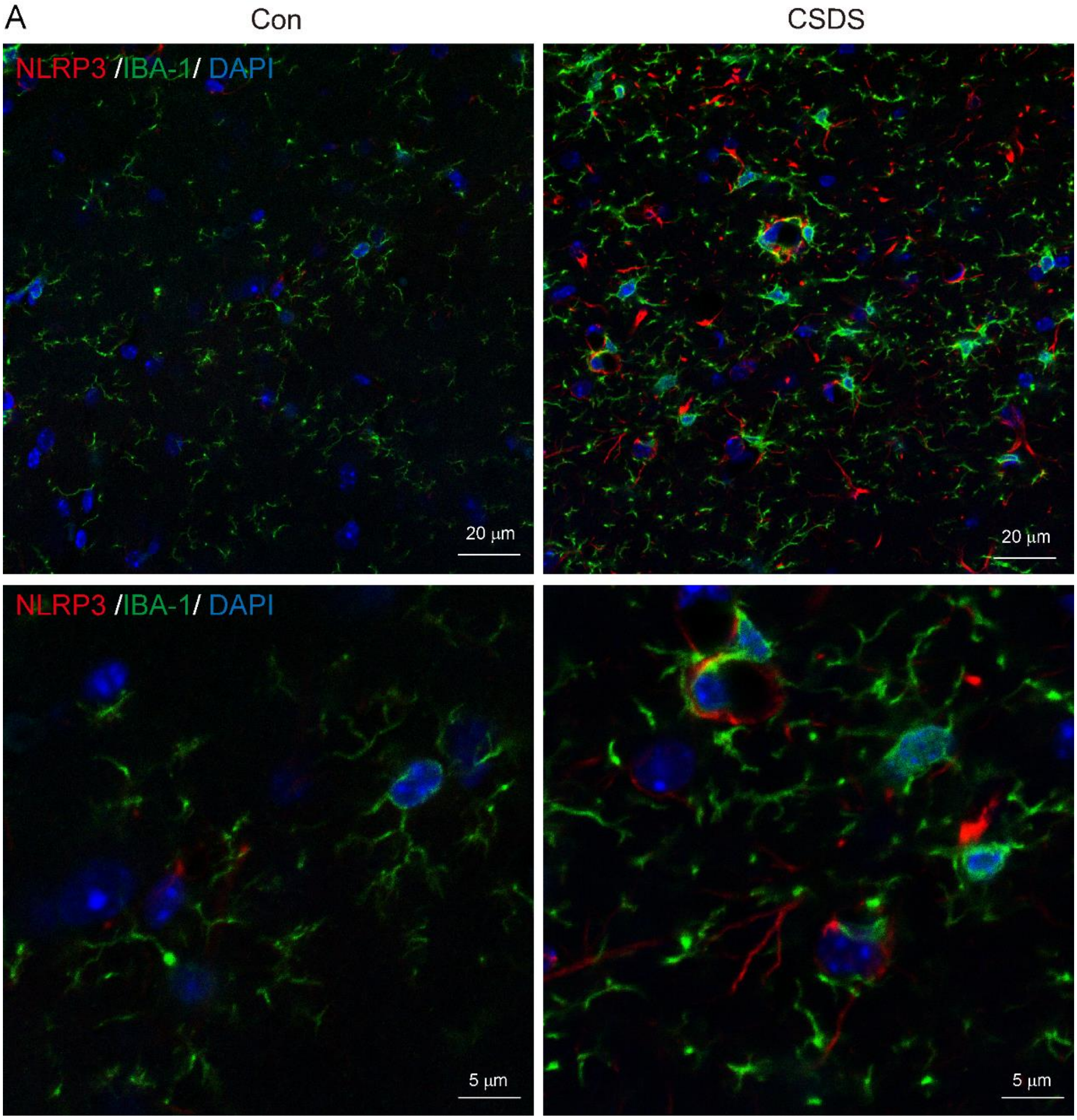

Figure S1 Representative double-immunostaining for NLRP3 (red) and microglia marker IBA-1 (green) in the hippocampus. (n=3 for each group).
